# COVID-19 vaccination in patients with primary immunodeficiencies: an international survey on patient vaccine hesitancy and self-reported adverse events

**DOI:** 10.3389/fimmu.2023.1166198

**Published:** 2023-04-18

**Authors:** Martine Pergent, Filomeen Haerynck, Levi Hoste, Ann Gardulf

**Affiliations:** ^1^ The International Patient Organisation for Primary Immunodeficiencies, Brussels, Belgium; ^2^ Primary Immune Deficiency Research Laboratory, Department of Internal Diseases and Pediatrics, Ghent University, Ghent, Belgium; ^3^ Department of Pediatric Pulmonology, Infectious Diseases and Immune Deficiency, Centre for Primary Immune Deficiency Ghent, Jeffrey Modell Diagnosis and Research Centre, Ghent University Hospital, Ghent, Belgium; ^4^ Department of Clinical Immunology, John Radcliffe Hospital, The International Nursing Group for Immunodeficiencies (INGID), Oxford University Hospitals NHSFT, Oxford, United Kingdom; ^5^ Division of Clinical Immunology and Transfusion Medicine, The Unit for Clinical Research, Department of Laboratory Medicine, Karolinska Institutet, Stockholm, Sweden; ^6^ Faculty of Social and Health Sciences, Department of Health and Nursing Sciences, Inland Norway University of Applied Sciences, Elverum, Norway

**Keywords:** COVID-19, SARS-CoV-2, vaccine hesitancy, vaccine adverse events, patient self-reported outcomes, quality of life, primary immunodeficiencies (PID), inborn errors of immunity (IEI)

## Abstract

**Introduction:**

The Sars-CoV-2 pandemic caused great concern for this novel virus among patients with primary immunodeficiency (PID) or inborn errors of immunity (IEI) and their families. When COVID-19 vaccination program started, no data existed on adverse events (AEs) in this particular patient population, nor if patients felt hesitancy being vaccinated.

**Objectives:**

To explore i) reasons for COVID-19 vaccination hesitancy, ii) the number and symptoms of AEs and their severity, durability and management.

**Method:**

The organisations International Patient Organisation for Primary Immunodeficiencies (IPOPI), European Society for Immunodeficiencies (ESID) and International Nursing Group for Immunodeficiencies (INGID) distributed a global self-administered online survey.

**Results:**

The survey was completed by 1317 patients (mean 47, range 12-100, years) from 40 countries. 41.7% of the patients denoted some hesitancy to COVID-19 vaccination, mainly having doubts about postvaccination protection related to their underlying PID and concerns about negative long-term effects. More women (22.6%) reported “very” or “pretty much” hesitancy compared to men (16.4%) (P<0.05). The most common systemic AEs were fatigue, muscle/body pain and headache, usually the same day or the day after the vaccination and lasting for 1-2 days. 27.8% of the respondents reported severe systemic AEs after any dose of COVID-19 vaccine. Only a minority (7.8%) of these patients visited a health-care professional and 20 patients (1.5%) were hospitalized or seen at emergency room without specifying subsequent admission at the hospital. Significantly more local and systemic AEs were reported after the second dose. No differences regarding AEs were observed across different PID subgroups or vaccine types.

**Conclusion:**

At the time of the survey, almost half of the patients reported having felt hesitancy to COVID-19 vaccination highlighting the importance and need of developing joint international guidelines and education programs about COVID-19 vaccination. The types of AEs were comparable to healthy controls, but more frequent AEs were reported. Clinical studies and prospective, detailed registration of AEs related to COVID-19 vaccines in this patient population is of great importance. It is crucial to elucidate whether there is a coincidental or causal association between COVID-19 vaccine and some severe systemic AEs. Our data do not contradict that patients with PID can be advised to be vaccinated against COVID-19, in accordance with applicable national guidelines.

## 1 Introduction

Primary immunodeficiencies (PIDs) or inborn errors of immunity (IEI) are a large and growing group of more than 485 rare and chronic disorders, characterized by defects in various parts of the immune system, leaving individuals affected with increased susceptibility for severe, persistent, unusual and/or recurrent infections, immune dysregulation and/or autoimmunity (1; 2). Most patients with PID, mainly those with predominantly antibody deficiencies (PAD), rely on lifelong immunoglobulin replacement therapy (3). Being both complex and diverse, the management of these conditions requires experienced medical and nursing specialists.

With the onset of the SARS-CoV-2 pandemic, the life of these patients and their families became even more difficult, fearing infection by a novel virus to which no specific treatment existed. The first population-based mass vaccination program started early December 2020 and offered a first prophylaxis against severe symptoms of COVID-19 (4). However, patients with PID or relatives were worried about COVID-19 vaccination as a) immunocompromised patients were not included in the different vaccination clinical trials, b) technologies of mRNA vaccines were not commonly known, c) mRNA COVID-19 vaccines were recommended for immunocompromised patients, some hesitated when they received another type of COVID-19 vaccine and d) patients with antibody deficiency had doubts about postvaccination protection. In addition to this, “immunodeficiency patients” were addressed as a “at risk group of patients”, an expression that encompasses very many diverse conditions far beyond PID. Moreover, prioritization guidelines varied according to different local health authorities and many contradictory information circulated, as well as fake news about COVID-19 vaccines. At the time of vaccination, no data existed on tolerance and efficacy in this particular patient population causing hesitancy to receive COVID-19 vaccines. Hence, the International Patient Organization for Primary Immunodeficiencies (IPOPI), The European Society for Immunodeficiencies (ESID) and the International Nursing Group for Immunodeficiencies (INGID) joined forces to set up an online patient survey aiming at collecting data on COVID-19 vaccination hesitancy and side effects. The objectives of this survey were to explore i) the reasons for hesitancy, and ii) the number and symptoms of adverse events when existing, their severity, durability and management in patients with PID or IEI.

## 2 Methods

### 2.1 The questionnaire

The survey was conducted through a self-administrated online questionnaire on SurveyMonkey (Momentive^®^, San Mateo, California, USA) from 1^st^ July to 30^th^ September 2021. The link to the questionnaire was distributed among IPOPI national members, inviting them to engage their patients with PIDs, or their informal caregivers, to take part to the survey. Invites were also shared on IPOPI’s global social medias. ESID and INGID also disseminated the link to the questionnaire in their respective networks so that physicians and nurses could encourage their patients to answer it. For the patients’ convenience the questionnaire was provided in French, Italian, Dutch, Danish, Russian, Swedish, in addition to English. From now on, the informal caregivers (e.g., parents) will also be included as “patients”.

The questionnaire included questions about four possible local symptoms at the injection site; redness, local heat, swelling and itching. For each symptom, the patient was asked to mark if they had experienced this symptom and, if so, whether it occurred after the first, second and/or third dose. In addition, for each symptom, the patient could indicate whether they had experienced the symptom as mild, moderate or severe. This self-assessment of severity was based on a definition published by the Federal Drug Administration (FDA) (5) and was presented to the patients in the questionnaire. The definition of AE severity is presented below under 3.4.

Correspondingly, the questionnaire asked about nine possible systemic symptoms; chills and fever, headache, joint pain, muscle/body pain, fatigue/tiredness, nausea/vomiting, diarrhea, abdominal pain, mouth or throat itching/tingling. when in time it had occurred in relation to given dose, and self-assessed severity.

The patients were also asked how any symptoms had resolved (without any action, self-medication or with help from health-care professionals, HPC). At the end of the questionnaire, it was also possible for the patients to report other symptoms, treatments, or anything else they considered important for this survey.

### 2.2 Inclusion criteria

The survey addressed the following patients:

- Fully vaccinated at the time of the survey, meaning two doses of Pfizer/BioNTech (BNT162b2), Moderna (mRNA-1273), Oxford-AstraZeneca (ChAdOx1), Sputnik V (Gam-COVID-Vac) or Covivac vaccine, or one dose for Johnson & Johnson (Ad26.COV2.S) vaccine; or
- Those who had received none or only one of two doses and with no intention of being vaccinated or getting the second dose.

The patients who were not vaccinated because they could not access the vaccine or those who had not yet finished their vaccination scheme were not allowed to complete the questionnaire.

### 2.3 Ethical considerations and data protection

Confidential information of the respondents (i.e. name, e-mail or IP addresses) were not collected and the answers are thereby anonymous. Confidentiality and electronic consent to participate were requested and by agreeing the respondents indicated having read the information about confidentiality and being voluntarily participating. The respondent also had the opportunity to ask questions to IPOPI e-mail address. The anonymized datasets were downloaded from SurveyMonkey. For analysis data were stored at user-restricted and password-protected servers at Ghent University Hospital.

### 2.4 Definition of hesitancy

Five modalities were proposed to describe patients’ hesitancy before receiving (or not willing to receive) the COVID-19 vaccine. Patients were asked if they were hesitating “Very much”, “Pretty much”, “Not much”, “Not at all”, “Don’t know”. Were considered as Hesitant patients who answered “Very Much” and Pretty much”.

### 2.5 Definition of adverse events

Adverse events (AEs) after vaccination were categorized based on the occurrence of local or systemic symptoms. The severity of AEs was graded by the respondents based on the Toxicity Grading Scale as published by the FDA: mild = symptoms do not prevent usual daily activities, moderate = symptoms limit normal daily activities and severe = symptoms make normal daily activities difficult or impossible (5).

### 2.6 Data cleaning and statistical analysis

Raw data was exported from SurveyMonkey and imported in Microsoft Excel (Microsoft Corporation^®^, Redmond, Washington, USA) and IBM SPSS Statistics (IBM, Armonk, New York, USA) for subsequent data cleaning, statistical analysis, and data visualization.

Cleaning of the data included the exclusion of inputs from patients declaring to have diagnoses that were not compatible with PID (n=4), including patients with type 1 diabetes mellitus, Churg-Strauss syndrome, asplenia and secondary immune deficiency after chemotherapy for CLL. Equally, the dataset was screened on double inputs from the same individuals (n=2). Patients were grouped according to the International Union of Immunological Societies (IUIS) diagnostic groups (1, 6).

For the comparison of categorical variables two-sided chi square testing of proportions was performed. To compare continuous variables independent T-testing of means was carried out. Possible differences in the denominators have occurred because of missing input. Significancy level was set to P<0.05. Bonferroni correction for multiple testing was applied when necessary (dividing the significance level by the number of hypotheses).

## 3 Results

### 3.1 Demographics

**Figure 1:**
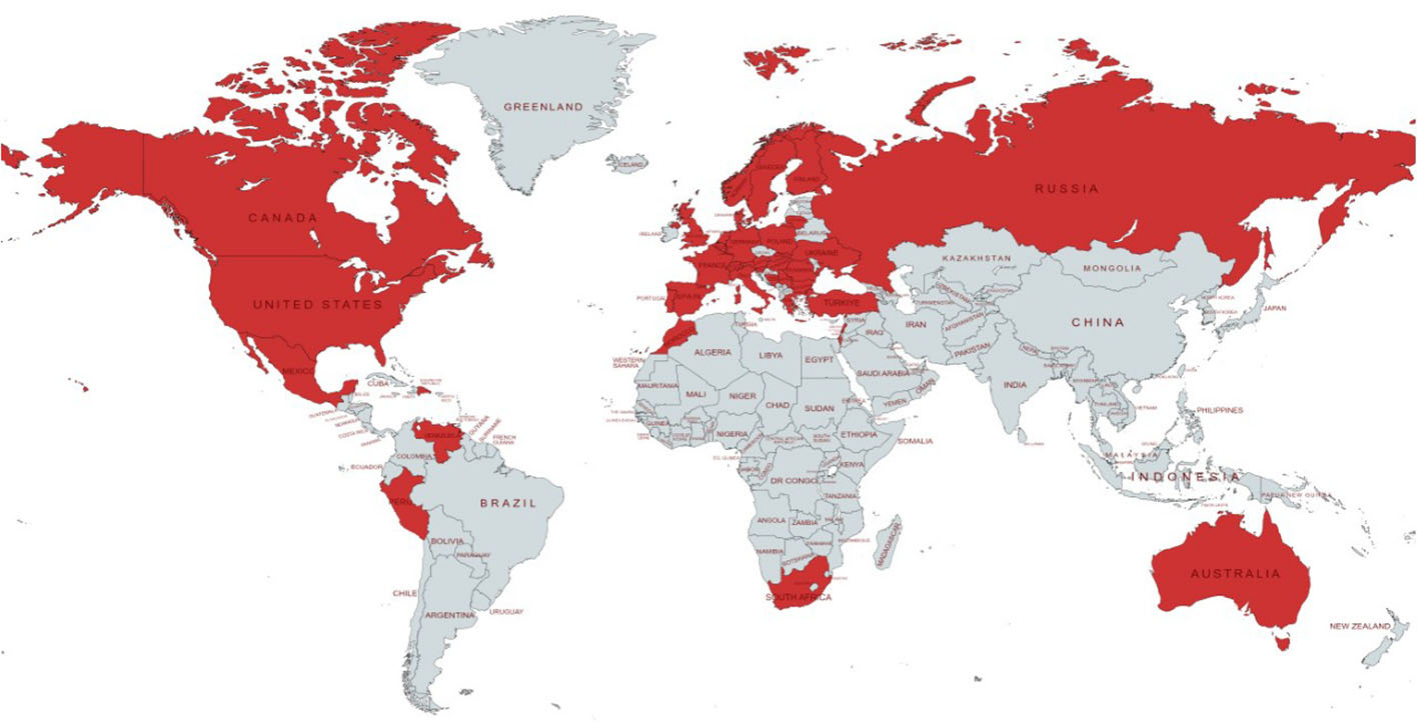
Geographical distribution of countries represented in the dataset. Each country with at least one patient with PID responding to the survey is colored in red.

The online survey was completed by 1324 unique patients diagnosed with PID or IEI. Data originated from 40 different countries, of which the majority were from Western European (813/1324; 61.4%) and North American countries (399/1324; 30.1%) (**Figure 1**).

The ages ranged from 5 to 100 years. Children younger than 12 years of age (n=7; 0.5%) were excluded from further analysis, since the survey was conducted at a time when COVID-19 vaccination was not yet approved for this age group. In total, 1317 patients with PID were included for the analysis. The study cohort had a mean age of 47 years [y] (standard deviation [SD] 16.6; **Table 1**). The majority of the respondents were female (971/1317; 73.7%) (**Table 1**). The female respondents were significantly older (mean 49y; SD 15.7) than their male counterparts (mean 41y; SD 17.9; 95% confidence interval of difference [-10.4;-6.4]).

**Table 1:** Demographics of the cohort of patients.

Gender	n	%
Female	971	73.7
Male	343	26.0
Other	3	0.2
Age*	**Years**	
Mean (SD)	47 (16.6)	
Range	12-100	
**Age groups**	**n**	**%**
12-17y	61	4.6
18-55y	771	58.6
>55y	484	36.8
**Total**	**1317**	**100.**

*Information about age was missing for 1 patient.

### 3.2 PID diagnoses and maintenance treatment

A large majority of the patients exhibited predominantly primary antibody deficiency (PAD 1140/1317; 86.6%), of which common variable immunodeficiency (CVID) was most frequent and was found in more than half of the total cohort (733/1317; 55.7%), followed by IgG subclass deficiencies (155/1317; 11.8%) and hypogammaglobulinemia (132/1317; 10.0%) (**Table 2**). PID affecting cellular and humoral immunity (31/1317; 2.4%) and congenital defects of phagocyte number or function (20/1317; 1.5%) were the second and third largest IUIS groups represented. Ninety-two patients (7.0%) had an unspecified PID, either because the patient was unaware of his/her specific diagnosis, or a formal diagnosis was not yet established, or had a diagnosis not yet included in the IUIS classification (mannose binding lectin (MBL) deficiency, n=10).

**Table 2:** IUIS* diagnostic groups categorizing the PID patients represented in the dataset.

Patients per PID diagnosis according to IUIS classification with at least 2 patients per diagnoses		
	n	%
**Predominantly antibody deficiencies**	**1140**	**86.6**
Common variable immune deficiency (CVID)	733	55.7
IgG subclass deficiencies	155	11.8
Hypogammaglobulinemia	132	10.0
Agammaglobulinemia (Bruton disease)	47	3.6
IgA deficiency	41	3.1
Specific polysaccharide antibody deficiency (SPAD)	21	1.6
Selective IgM deficiency	3	0.2
Other	2	0.2
Unclassified antibody deficiency	6	0.5
**Immunodeficiencies affecting cellular and humoral immunity**	**31**	**2.4**
Severe combined immunodeficiency (SCID)	13	1.0
Combined immunodeficiency (CID)	11	0.8
Hyper IgM syndrome	7	0.5
**Congenital defects of phagocyte number or function**	**20**	**1.5**
Chronic granulomatous disease (CGD)	14	1.1
Congenital neutropenia	4	0.3
Other	2	0.2
**Combined immunodeficiencies with associated or syndromic features**	**14**	**1.1**
Hyper IgE syndrome (HIES)	5	0.4
Di George syndrome	4	0.3
Wiskott Aldrich syndrome	2	0.2
Other	3	0.2
**Diseases of immune dysregulation**	**10**	**0.8**
Autoimmune lymphoproliferative syndrome (ALPS)	3	0.2
CTLA4 deficiency	3	0.2
Immune dysregulation polyendocrinopathy enteropathy X-linked (IPEX)	2	0.2
Other	2	0.2
**Defects in intrinsic and innate immunity**	**6**	**0.5**
STAT1 gain-of-function (GOF)	2	0.2
Warts, hypogammaglobulinemia, infections and myelokathexis (WHIM) syndrome	2	0.2
Other	2	0.2
**Complement deficiencies**	**3**	**0.2**
**Autoinflammatory disorders**	**1**	**0.1**
**Unspecified and/or not in IUIS classification**	**92**	**7.0**
Unspecified PID according to patients	76	5.8
MBL deficiency	10	0.8
Other	6	0.5

*Update on the classification from the International Union of Immunological Societies (IUIS) Expert Committee.

The bold values represent the sums of the different diseases that are included in each section.

Compatible with many patients suffering from PAD, 1061/1317 (80.6%) of the cohort was on life-long immunoglobulin replacement therapy (IRT). Immune suppressants or immunomodulatory drugs were less frequently used (154/1317; 11.7%). Immunosuppressive drugs (e.g., sirolimus, mycophenolate mofetil, cyclosporine), besides high dose steroids were prescribed in 77/1317 (5.8%) to the patients. Only a small minority of patients used a combination treatment of multiple immune suppressants or immunomodulatory drugs (n=11). IRT was most frequently used in patients with PAD (969/1140; 85%) and immunodeficiencies affecting cellular and humoral immunity (26/31; 84%). The highest proportion of patients on immune suppressive or immunomodulating therapy were those with immunodeficiencies affecting cellular and humoral immunity (9/31; 41%).

### 3.3 COVID-19 vaccination

Much heterogeneity was reported by the patients/informal caregivers concerning the prioritization for COVID-19 vaccination to individuals with PID. Although many countries used some prioritization guidelines, patients with PID were mostly not prioritized from the very beginning of the national vaccination campaigns (505/1310; 38.5%). Prioritization occurred most frequent in Western European countries with three quarters of respondents declaring prioritization from the beginning (33.2%) or at a later timepoint (40.9%). One in 5 patients declared that patients with PID were not prioritized according to local guidelines (292/1310; 22.3%), especially in North American (39.8%) and Eastern European countries (31.3%).

**Figure 2:**
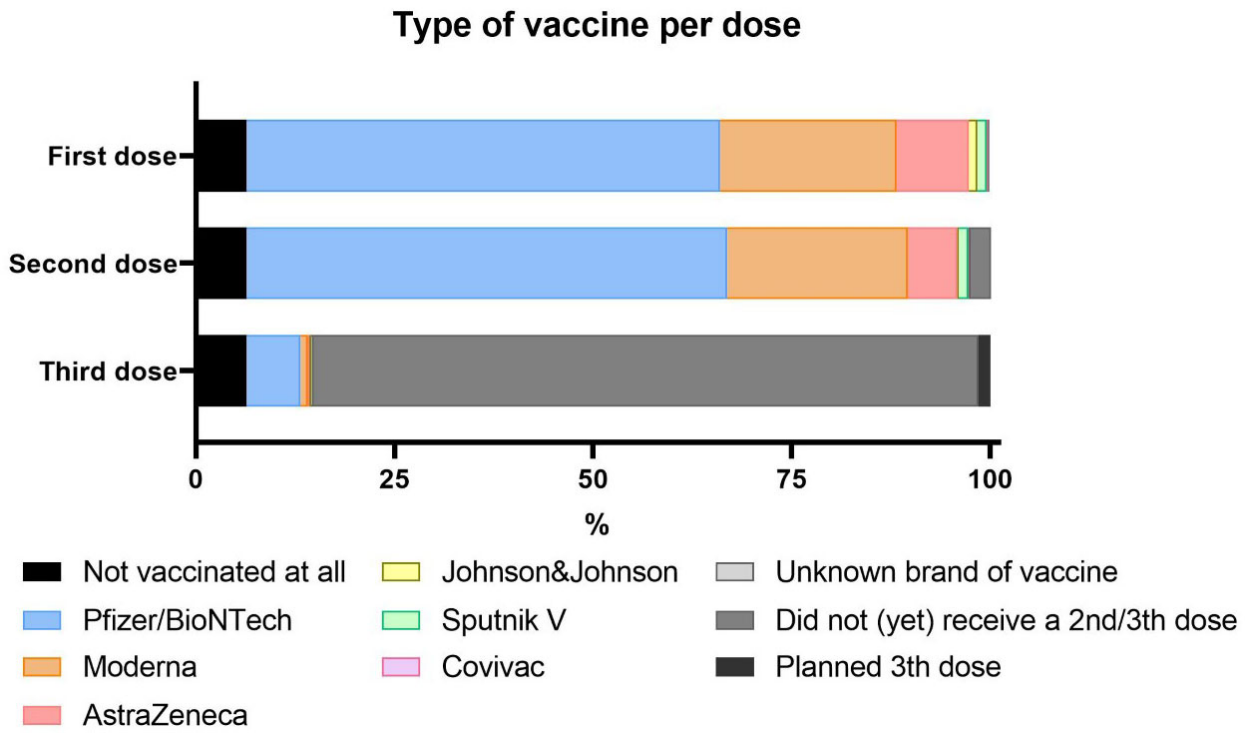
Type of COVID-19 vaccine per dose used in patients with PID that responded to the survey.

Most participants were fully vaccinated (1210/1317; 91.8%). In line with the distribution and availability of vaccines, the majority of patients had received a mRNA based vaccine (81.9% of first doses, 83.3% of second doses), mostly vaccines from Pfizer/BioNTech (59.7% and 60.5%, respectively). Booster campaigns were not yet initiated at the time of the survey and only 8.6% (108/1259) of the cohort had received a third dose. (**Figure 2**). No proportional difference in vaccination status was found when comparing demographics or the underlying IUIS diagnostic groups.

In terms of underlying PID groups, there were no significant differences in the proportions of vaccine brands. In contrast, some geographical differences were reported; relatively more AstraZeneca and Pfizer in Western Europe, more Moderna and Johnson and Johnson in North America, and more Sputnik V in Eastern Europe. Patients younger than 55 years of age received more frequently Pfizer/BioNTech and AstraZeneca (data not shown).

### 3.4 COVID-19 vaccination hesitancy

A large proportion (541/1296; 41.7%) denoted some hesitancy to COVID-19 vaccination including 11.3% and 10.0% of patients reporting “very much” and “pretty much” hesitancy, respectively. Mainly women (216/955; 22.6%) reported “very” or “pretty much” hesitancy as compared to the men (55/335; 16.4%) (P<0.05). Furthermore, a significantly larger proportion of the patients living in Eastern Europe (25/65; 38.5%) reported more “very” or “pretty much” hesitancy as compared to North America (82/392; 20.9%) (P<0.05) or Western Europe (153/795; 19.2%) (P=0.01).

Ninety-four patients (94/1317; 7,1%) did not want to have a COVID-19 vaccine at all, and 14/1317 (1.1%) reported that they received one dose but did not want to obtain a following dose. Although this concerned small proportions of the entire cohort, vaccine reluctance was significantly larger in African (4/8; 50.0%) and Eastern European countries (13/67; 19.4%) as compared to North American (38/398; 9.5%) and Western European countries (36/810; 4.4%) (P<0.05). There were no proportional differences in vaccination hesitancy when comparing underlying IUIS subgroups.

148 patients (148/1317; 11.2%) reported a SARS-CoV-2 infection prior to COVID-vaccination. Willingness to be vaccinated was significantly higher among patients with PID without prior SARS-CoV-2 infection (1080/1154; 93.6%), as compared to patients who had experienced SARS-CoV-2 infection before the vaccination (128/148; 86.5%) (P<0.01).

**Figure 3:**
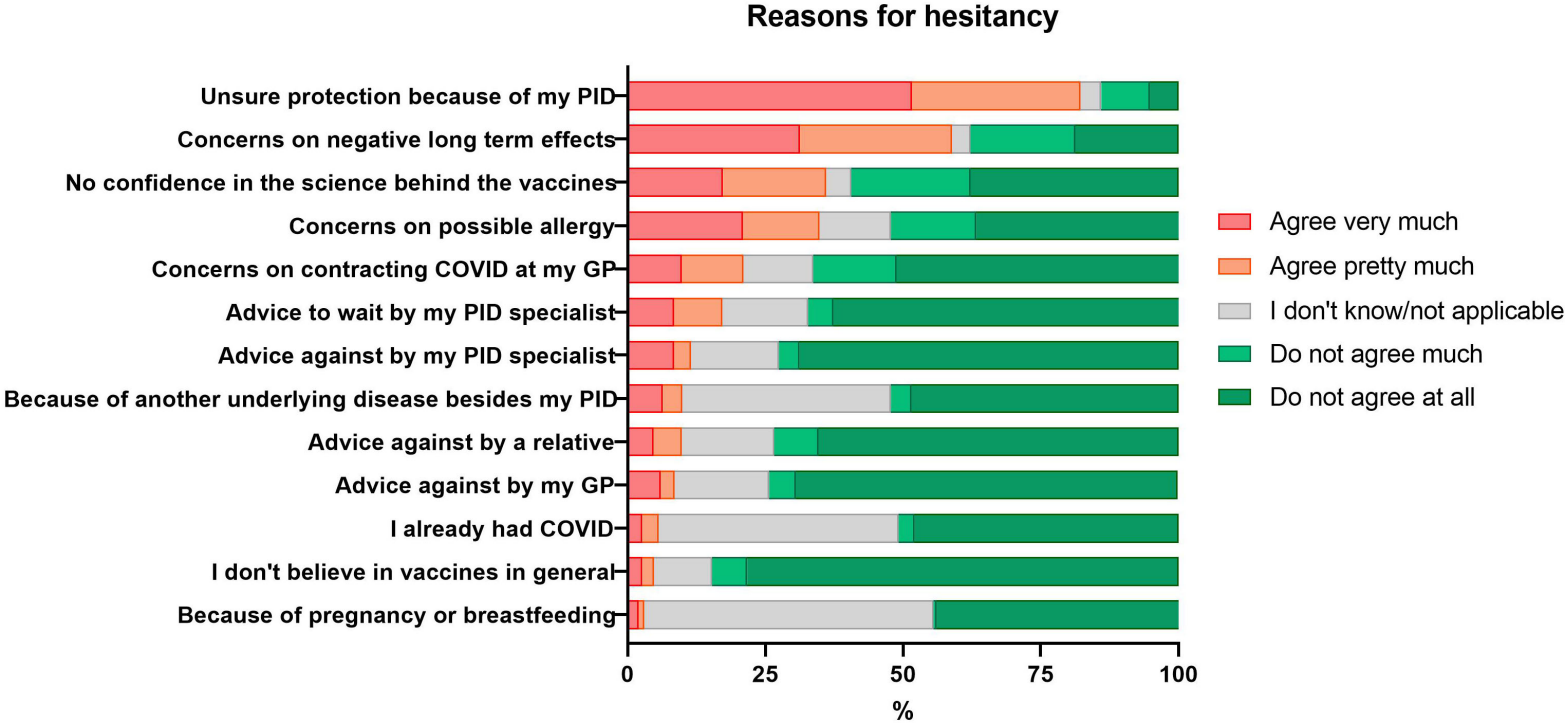
Self-reported reasons for hesitancy to get vaccinated against COVID-19 in patients with PID.

The most common reasons given by the patients who reported “very” and “pretty much” hesitancy included doubts about postvaccination protection because of the underlying PID (426/519; 82.1%), concerns about negative long-term effects (300/510; 58.8%), absence of confidence in the science behind the current vaccines (184/515; 35.7%), and concerns on possible allergies (179/513; 34.9%). The apprehension seemed to be COVID-19 vaccine specific, as only 24/511 (4.7%) of the patients stated that they did not believe in vaccines in general (**Figure 3**).

Almost one-fifth of the respondents (86/510; 16.9%) were hesitant because their PID specialist had recommended them to wait with the vaccination against COVID-19 or not to initiate a COVID-19 vaccination process (57/509; 11.2%). The advice against vaccination was more frequently reported by patients with congenital defect of phagocyte number or function (3/7; 42.9%) and immune dysregulation (2/5; 40%), as compared to other PID diagnostic groups. The advice to wait was more frequently reported by patients with immune suppressant other than steroids (10/28; 35.7%) as compared to those without this treatment (75/479; 15.7%) (P<0.01). The advice to wait was more often reported by patients from Eastern Europe (14/26; 53.8%) as compared to North America (31/146; 21.2%) and Western Europe (38/317; 12.0%) (P<0.01 and P<0.001, respectively).

COVID-19 vaccine hesitancy was significantly more frequent stated by patients who had experienced at least one moderate (138/757; 18.2%) or one severe reaction (84/339; 24.8%) after any dose, and in patients with at least one local (28/104; 26.9%) or systemic (46/206; 22.3%) severe AEs, as compared to the proportion without such AEs (43/386; 11.1%, 97/804; 12.1%, 143/1010; 14.2%, and 102/826; 12.3%, respectively) (data not shown).

### 3.5 Adverse events

In total, 1069 patients (93.1%) described at least one local or systemic mild AEs after any dose. Mild (1012/1148; 88.2%) and moderate adverse events (5735/1148; 64%) were most frequently experienced whereas severe AEs were less common (347/1148; 30.2%). No significant differences regarding AEs in terms of underlying PID were found.

Patients (72/106; 68%) who had been infected with Sars-CoV-2 prior to vaccination reported moderate AE or severe AE, compared to those (548/1006; 54%) who had no COVID-19 prior infection, but significantly only after the second dose (P=0,008). AEs after the first dose do not show any significant difference. AEs are also more pronounced among those infected who reported a severe disease course (89% - 33/37) and those infected who reported no severe disease course (57% - 39/69) (P=0,001).

#### 3.5.1 Local adverse events

**Figure 4:**
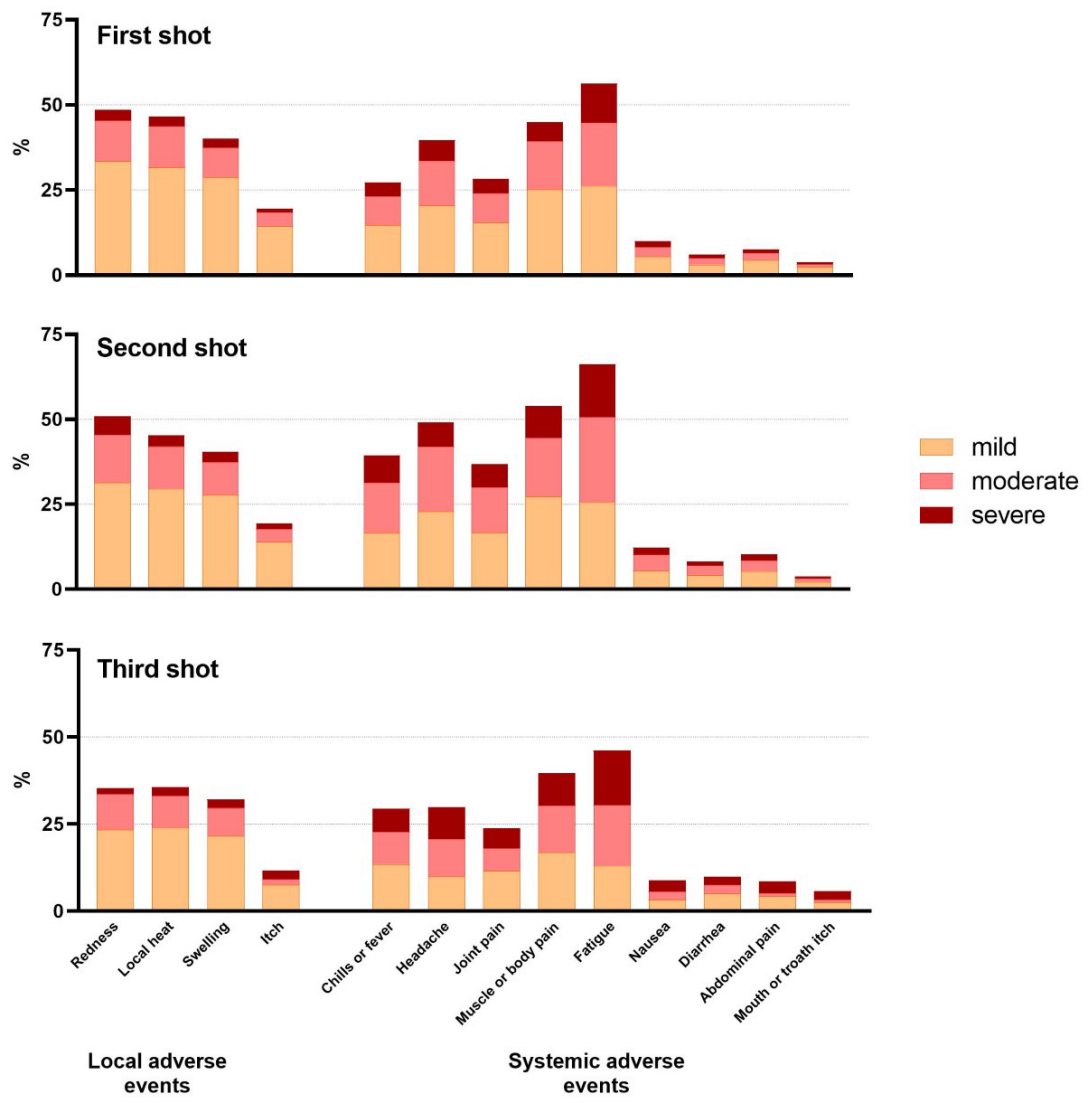
Percentages of patients with PID reporting local and systemic adverse events after COVID-19 vaccination.

Two third of respondents reported at least one local reaction at the site of the injection after vaccination (882/1153; 76.5%), the most common being redness, local heat and swelling. The local AEs were mostly experienced as mild (69.4%) (**Figure 4**).

Patients reported significantly more local AEs after the second vaccine dose; 25.7% (286/1115) moderate and 8.3% (92/1115) severe local AEs, as opposed to 22.4% (258/1150) moderate and 6.1% (70/1150) severe after the first vaccine dose (P<0.05). In contrast, mild local AEs were similar after first and second vaccine dose (**Table 3**).

**Table 3:** Any type of local and/or systemic adverse event (AE) after first, second and third dose of COVID-19 vaccine categorized according to mild, moderate, severe, or at least mild AEs.

	Mild		Moderate		Severe		Any severity (at least mild)		Total count
**First dose**	n	%	n	%	n	%	n	%	n
Any local AE	676	58.8	258	22.4	70	6.1	783	68.1	1150
Any systemic AE	652	56.9	408	35.6	180	15.7	795	69.4	1145
Any local or systemic	879	76.9	502	43.9	199	17.4	950	83.1	1143
**Second dose**									
Any local	643	57.7	286	25.7	92	8.3	775	69.5	1115
Any systemic	681	61.2	514	46.2	231	20.8	872	78.3	1113
Any local or systemic	867	78.3	587	53.0	250	22.6	960	86.7	1107
**Third dose**									
Any local	57	31.5	20	11.0	5	2.8	69	38.1	181
Any systemic	49	23.9	36	17.6	21	10.2	66	32.2	205
Any local or systemic	71	58.2	40	32.8	22	18.0	77	63.1	122
**Any dose**									
Any local	800	69.4	385	33.4	134	11.6	882	76.5	1153
Any systemic	865	75.3	663	57.8	319	27.8	993	86.5	1148
Any local or systemic	1012	88.2	735	64.0	347	30.2	1069	93.1	1148

n=number of patients reporting an adverse event, %=percentage of total count.

Despite. a lower number of patients who received a booster dose, for local AE the proportion of local AE after the third dose is significantly lower when looking at “at least mild” (57; 31,5%), “at least moderate” (20; 11,0%) or “severe”(5; 2,8%) AE. For this we analysed(Chi-square) the proportion of AE after the third dose as opposed to the proportion of AE after the first and second dose combined (**Table 3**).

#### 3.5.2 Systemic adverse events

86.5% (993/1148) of the patients mentioned at least one systemic AE (**Table 3**) after any dose, mostly reported as mild manifestations (75.3%). Fatigue, muscle or body pain and headache were most frequently indicated (**Figure 4**). Half of the patients (663/1148; 57.8%) reported moderate systemic AEs and one third (319/1148; 27.8%) severe AEs after any vaccine dose (**Table 3**).

Systemic AEs were more often perceived after the second dose in comparison to the first. After the second dose, mild (681/1113; 61.2%), moderate (514/1113; 46.2%) and severe (231/1113; 20.8%) systemic AEs were reported as opposed to after the first dose; (652/1145; 56.9%) (P<0.0001), (408/1145; 35.6%) (P<0.0001) and (180/1145; 15.7%) (P<0.01) (**Table 3**).

Patients reporting severe systemic AEs were significantly more frequently female (255/313, 81.5%) (P<0.001) and between 18 and 55 years of age (217/312; 69.6%) (P<0.05). The most reported moderate and severe systemic AEs encompassed fatigue, muscle pain, headache and fever.

As per the third dose, the proportion of systemic AE is significantly lower when looking at “at least mild” (49; 23,9%), “at least moderate” (36; 17,6%) or “severe” (21; 10,2%) AE. (Chi square analysis) (**Table 3**).

#### 3.5.3 Duration and treatment of local and systemic AEs

In most cases, systemic or local AEs occurred within the same day or one day after vaccination (814/1140; 71.4%) and lasted no longer than 2 days (667/995; 67.0%). However, 77 (7.7%) of the patients declared to have experienced symptoms that had lasted more than a week. Most of these AEs resolved without any action or with self-medication (880/954; 92.2%) (**Table 4**).

**Table 4:** Reported onset and duration of severe local or systemic AEs, actions taken and resolving of symptoms.

		n	%
**Onset of severe adverse events**	The same day as the vaccination	259	22.7
	The day after the vaccination	555	48.7
	2 days after the vaccination	62	5.4
	3-6 days after the vaccination	42	3.7
	1 week or more after the vaccination	22	1.9
	Other	10	0.9
**Duration of severe adverse events**	1 day	381	38.3
	2 days	286	28.7
	3 days	128	12.9
	4-7 days	123	12.3
	More than a week	77	7.7
**Action taken by the patients**	No action taken	433	45.6
	Self-medication	447	47.1
	Physician visit	54	5.7
	Emergency room visit	4	0.5
	Hospitalization	10	1.0
	Unspecified urgent care	6	0.5
**Reported resolving of severe adverse events**	Self-resolved fully without treatment	499	52.9
	Resolved fully with treatment	385	40.8
	Still generalized symptoms	60	6.4

Only a minority (74/954; 7.8%) experienced local or systemic AEs that made them require advice from HCPs. In total, 20/1317 patients (1.5%) reported they needed to be hospitalized or seen at emergency room without specifying subsequent admission at the hospital. The symptoms reported by these 20 patients, mainly women (18/20, median age 46 y [16y-68y]), are presented in **Table 5**. Severe adverse events needing advice from HCPs were reported as well after the first dose (9/20) as after the second dose of vaccination (11/20). Severe local symptoms or local infection was observed in only 2 of them. The clinical manifestations of severe systemic AEs needing HCP advice were heterogeneous. Severe gastro-intestinal symptoms (4/20) including diarrhea, severe abdominal pain and nausea were mainly treated with IV fluid. One patient received IV corticosteroids for severe diarrhea. A 29-years old male reported acute myocarditis after the second dose. Five patients recorded central nervous symptoms including severe headache in two respondents, one patient revealing intracranial haemorrhage with left side paralysis, one person with vertigo and another patient experienced a flair of dysautonomia syndrome. All neurological complications, except severe headache, were still present at the time of the survey. Thrombocytopenia was described by 3 patients requiring splenectomy in one of them unclear of this was related to the underlying PID or secondary to COVID-19 vaccine. Fully resolved anemia was stated by one patient. Remarkable, half of them mentioned that the severe systemic adverse events were still ongoing at the time of the study (10/20). Only one patient presented with severe allergic reaction needing adrenalin (**Table 5**).

**Table 5:** Clinical details of patients reporting severe local or systemic adverse events needing emergency room visits, hospitalizations after COVID vaccination.

	Country	Gender	Age	PID	Therapy	Prior COVID	1st dose	2^nd^ dose	3rd dose	Self-reported reasons for seeking health-care	Timing	Resolution (at time of survey)
ER	USA	M	29	Selective IgM Deficiency		Yes, moderate	Moderna	Moderna	–	Myocarditis, ongoing mild symptoms after 2 months	After second dose	Ongoing
ER	Norway	F	47	IgG Subclass deficiencies	Igs		Pfizer	Pfizer	–	Local infection at injection site	After second dose	Fully resolved
ER	UK	F	61	CVID	Igs		Pfizer	Pfizer	–	Severe headache	After second dose	Fully resolved
ER	USA	F	69	CVID	Igs		Moderna	–	–	Allergic reaction needing adrenalin	After first dose	Fully resolved
Hosp	USA	F	42	IgG subclass deficiency	–	Yes, severe	Pfizer	Pfizer	Pfizer	Answered severe to all questions	NA	Ongoing
Hosp	Sweden	F	54	CVID	Igs	–	AZ	–	–	Thrombocytopenia	After first dose	Ongoing
Hosp	USA	F	67	IgA deficiency	–	–	Moderna	Moderna	–	Severe vertigo	After first dose	Ongoing
Hosp	Spain	F	68	Unclassified	Igs	Yes, severe	Pfizer	Pfizer	–	Gastro-intestinal symptoms (steatorrea, lipase elevation,…)	After second dose	Ongoing
Hosp	Australia	F	63	CVID	–	–	AZ	–	–	Intracranial haemorrhage, left sided paralysis	After first dose	Ongoing
Hosp	Belgium	F	18	Unclassified	Igs	Yes, severe	Pfizer	Pfizer	Pfizer	Anemia	After second dose	Fully resolved
Hosp	France	F	33	CVID	Igs	–	Pfizer	–	–	Severe abdominal pain, headache, fatigue	After first dose	Fully resolved
Hosp	Sweden	F	68	IgG Subclass deficiencies	Igs	–	Pfizer	Pfizer	Pfizer	Severe local reaction	After second dose	Unknown
Hosp	USA	F	62	CVID	Igs, steroids	Yes, severe	J&J	–	–	Neurological symptoms	After first dose	Ongoing
Hosp	France	F	33	CVID	Igs, steroids, biologicals	–	Pfizer	–	–	Urinary retention	After first dose	Fully resolved
Other	Romania	F	16	CVID	Igs, other IS	–	Pfizer	Pfizer	–	Thrombocytopenia	After second dose	Fully resolved
Other	USA	F	60	SCID	Igs, steroids	Yes, moderate symptoms	Moderna	Moderna	–	Severe diarrhea, IV steroids administered	After second dose	Ongoing
Other	Belgium	F	21	Roifman syndrome	Igs	–	Pfizer	Pfizer	–	Severe abdominal pain, nausea, headache and fatigue, IV fluids ()? and paracetamol	After second dose	Fully resolved
Other	USA	M	45	CVID	Igs	–	Pfizer	Pfizer	**-**	Flair of dysautonomia syndrome, requiring IV ivabradine	After second dose	Ongoing
Other	USA	F	63	CVID	Igs, biologicals	Yes, severe symptoms	Moderna	Moderna	–	Severe local reaction and severe headache and fatigue, IV steroids	After second dose	Fully resolved
Other	Sweden	F	54	CVID	Igs	–	AZ	–	–	Thrombocytopenia, needing splenectomy and IVIG	After first dose	Ongoing

## 4 Discussion

Our online patient survey reports on patients with PID (n=1317) facing infection with SARS-CoV-2 and their experiences with vaccination against COVID-19. The survey specifically focused on reasons for possible vaccine hesitation and AE incidence, severity, durability and management. At the time of the survey, patients who had received two doses of vaccine against COVID-19 were considered as fully vaccinated, but some were already advised by their physician to get a third dose. Because the latter group was small, we focused the analysis on self-reported AE after the first and second vaccine dose.

With three-quarter of respondents being female, a sex bias was present. Although such imbalance is described in questionnaires that touch upon health-related quality of life, it is desirable for more men to share their views and experiences (7). Since females in our cohort were older and reported more systemic AE than their male counterparts, this might influence interpretation of the overall dataset. Most respondents were from Europe and North American countries. Our conclusions thus mostly reflect data from these two regions, including their vaccines available, given the small samples from other world regions. Unsurprisingly, the majority of respondents were affected by predominant antibody deficiencies, which constitutes the largest group of patients within PIDs (8).

We graded the severity of AEs based on the Toxicity Grading Scale published by FDA (5). This allowed us to categorize the reported AEs based on their severity, which by the FDA’s scale means mild, moderate, or severe. Clinical COVID-19 vaccine trials in healthy controls mostly used four gradations and defined severe (grade 3) as limitation of activity and requirement of medical interventions and potentially life-threatening (grade 4) requiring ER or admission to hospital (9, 10).

As this concerns a survey with self-reported data, the response alternatives must be perceived as adequate and easy to understand by the respondent. However, the results must be interpreted with caution as they cannot be objectively verified.

About 41% of the patients denoted some hesitancy to COVID-19 vaccinations, which was mostly driven by unsure protection because of the underlying PID (82%) or doubts on possible long-term effects (59%). Recently, another survey on 370 Canadian patients with PID indicated that 18.4% of them were somewhat or very unlikely, undecided, or not planning to get vaccinated with PID. Important to notice is that most of the patients in the Canadian study were not yet vaccinated against COVID-19 (11) A recent Polish study, studying 114 PID patients and 36 patients with auto-inflammatory diseases, indicates that 80% was vaccinated. About 41% of the patients denoted some hesitancy to COVID-19 vaccinations, which was mostly driven by unsure protection because of the underlying PID (82%) or doubts on possible long-term effects (59%), similar findings as in our survey. In addition, a strong correlation was found between hesitancy and primary or vocational education. The patients that had decided to get vaccinated against COVID-19 had a fear of getting sick with COVID-19 and reported that they felt it was the correct decision and that they had been supported in their decision from experts and relatives (12).

A significantly larger proportion of female respondents reported hesitancy as compared to men. The same result was seen in the Canadian study with 75.4% of the respondent being women (11). No proportional difference in vaccination status was found when comparing demographics or IUIS diagnostic groups.

The proportion of patients with PID reporting vaccine refusal or discontinuation was low (8.2%). Comparing the proportion of hesitancy with that of the general population is challenging because of substantial differences depending on the country and national guidelines (13) Equally, because the respondents chose to participate, a reporting bias might skew our data.

To take lessons for the future, we aimed to understand reasons behind hesitancy or refusal of the vaccine. Patients’ hesitancy seemed to be COVID-19 specific, as only 4.7% stated that they did not believe in vaccines in general. Hesitancy was more frequently related to the respondent’s immunodeficiency, such as uncertainty about protection provided by the vaccine and concerns on allergic reactions. Secondly, long-term harmful effects and lack of trust in novel mRNA vaccine technology directed the patients’ hesitancy. Fewer patients declared external reasons, including advice against or to postpone vaccination by physicians and/or relatives.

Similar results were seen in the Canadian study where uncertainty on the immune response and long-term AE were the most cited reasons (11). Our data on hesitancy are similar as reported by other immunocompromised patients, i.e. systemic rheumatic disease (12, 14). We hypothesize that advice to wait was most given awaiting further evidence on efficacy and safety of these novel vaccines in patients with PID. Moreover, the pandemic time was a period where people could see the science in the making, resulting in uncertainty among patients linked to the situation itself, to accompanying debates in the community and to heterogeneity of practices in different regions. To tackle this problem, patient organizations gave several online webinars to provide objective information with focus on COVID-19 and PID. Another issue contributing to this uncertainty is that the generic term “immunocompromised persons” concerns a very heterogeneous group of patients of which persons with PID is only a small part. Furthermore, PIDs are very diverse conditions emphasizing the importance of well-informed health-care professionals and international guidelines.

This knowledge and the observations regarding patient hesitancy highlight the necessity of education of physicians and nurses about the efficacy and safety of COVID-19 vaccination to patients with PID, which is also pointed out in other studies (11, 12; Squire, Avni, and Joshi, n.d.; 15).

Two third of the respondents (76.5%) stated redness, local heat and/or swelling at the site of the intramuscular injection. Most local AEs were reported as mild similar to observations in COVID-19 vaccine clinical trials with healthy individuals (9, 10, 16). Only two patients reported severe local reactions that required help from health care professionals. To prevent unnecessary worries, it is important that patients are well informed on expected local reactions, e.g., by nurses that administer the vaccines.

In total 86.5% denoted at least one systemic adverse reaction after any dose of vaccination, mostly mild manifestations (75.3%). Half of the patients (57.8%) reported moderate systemic AEs i.e. symptom(s) that limited normal daily activities. One third (27.8%) presented severe AEs i.e symptom(s) making normal daily activities difficult or impossible after any vaccine dose. This contrasts with studies including healthy controls where severe AEs have been very rare (9, 10, 17). In studies including adult patients with PID the proportion of patients observed to present with severe AEs differs between 0-15% (13; 18; 19, 20). The Polish study reported that AEs had occurred in 35.1% of the patients with primary immunodeficiencies, however the severity of the AEs is not mentioned.

Up to December 2022, approximately 104,500 reports on suspected adverse reactions has been sent by HCPs, or single individuals, to the Swedish Medical Products Agency (Swedish MPA) “Https://Www.Lakemedelsverket.Se/En/Coronavirus/Covid-19-Vaccine/Reported-Suspected-Adverse-Reactions-Corona-Vaccines”. Fatigue is the most frequently reported symptom, followed by muscle and/or body pain and headache similar to other studies (9, 10).

Systemic adverse events were more often reported after the second dose which is consistent with clinical trials in healthy controls (9, 10) and other reports on COVID-19 vaccination in patients with PID respectively (17, 19). Most importantly, no significant differences in terms of underlying PID were found.

The majority of systemic AEs occurred the same day or the day after vaccination and lasted no longer than 2 days which is comparable with clinical trials of COVID-19 vaccines (9, 16, 20). Most AEs required only self-medication. However, 7.7% of patients reported symptoms that lasted more than a week in contrast to other clinical trials reporting resolution of most symptoms within one week (9).

In total, 7.8% patients reported systemic or local site reactions where they felt that they required help from HCPs. Notably, 20 patients (1.3%) were hospitalized or had to seek urgent care due to severe AEs. Although possibly related to a sampling bias, a majority of these (18/20, 90%) were females. Most of the severe AEs where patients who needed help at an ER or were admitted to hospital presented with very serious symptoms from a medical point of view. The four patients with severe gastro-intestinal symptoms all suffered from CVID or (S)CID which is known to be associated with enteropathy. Flare of enteropathy after COVID-19 vaccination has been previously reported in one patient with PID (21). Among our patients a 29-years old male reported acute myocarditis after the second dose of Moderna vaccine. Post-mRNA vaccination myocarditis is seen predominantly in young males within a few days after their second dose of vaccination. While the pathophysiology of myocarditis is not well known, its prognosis is good as all reported patients have recovered (22). Five patients recorded central nervous symptoms ranging from unspecific neurological symptoms, severe headache, severe vertigo to intracranial haemorrhage with left side paralysis (all females, age range 61-67 years) and a flair of dysautonomia syndrome (male, 45 years). All neurological complications, except severe headache, were still present at the time of the survey. We could not obtain more detailed information on the cause of the central nervous symptoms because respondents were anonymized. Cerebral venous sinus thrombosis with thrombocytopenia, especially in women, younger than 60 years is a documented post vaccination complication requiring hospitalization (23).Thrombocytopenia was described by 3 patients (all females, 2 patients receiving Oxford-AstraZeneca (ChAdOx1), age range 16-54 years), one requiring splenectomy (54 years of age). Similar, thrombocytopenia is a known AE post-COVID-19 vaccinations in healthy controls more frequently seen in women and after Oxford-AstraZeneca (ChAdOx1) but does not suggest a safety concern (24, 25). Fully resolved anemia was stated by one patient. Autoimmune cytopenia after COVID-19 vaccination has also been observed in healthy controls (23).Importantly, autoimmune cytopenia is a common presentation of CVID because of immune dysregulation, also beyond vaccination (26). Half of our patients with cytopenia mentioned that severe systemic adverse events were still ongoing at the time of the study.

Of particular interest is, that of all 1317 patients, only one reported an allergic reaction requiring treatment. The risk of severe allergic reactions thus appears to be low in patients with PID. This is important information to discuss with the patient and informal caregivers as about 40% of the patients in our study reported that their hesitancy to be vaccinated against COVID-19 was based on fear of having an allergic reaction.

Our data show that patients with PID experience local adverse events comparable with the general population. Severe AEs after the first dose were more commonly reported by our patients with PID compared to studies including healthy controls (9, 10, 20) and somewhat higher than other clinical studies including patients with PID (13; Squire and Joshi; 20) or in line with one of them (19). To draw well-founded conclusions, a case-by-case analysis of severe systemic and long-lasting AEs should occur which is not achievable through self-reported surveys. Reports on larger groups of PID are crucial to elucidate whether the association severe AEs and COVID-19 vaccination is coincidental and more likely a manifestation of the underlying immune deficiency or causal. Notwithstanding these limitations, this study provides very useful information on reasons for hesitancy among adult patients with PID and self-reported AE regarding incidence, severity, durability, and management.

### 4.1 Limitations and strength

We underline that all symptoms were self-reported by patients and were neither confirmed neither disproved by the treating physician because of the COVID-19 vaccine. Survey participants chose to complete the survey and a selection bias could be present as well. Still, to include patient-reported outcomes in clinical studies are considered to be very important and is today recommended in research literature (27).

One should be cautious to compare our data with incidences in for example randomized controlled clinical trials. The sample of patients does not allow us to say whether these manifestations occur more in one or more PID category. Several reports on immune reactivity to COVID-19 vaccines in individuals with various PID did not report higher numbers of systemic adverse events as compared to healthy controls (19, 26, 18, 27).

We also want to highlight the fact that the number of participants vaccinated with Pfizer and Moderna was much higher than with the other vaccines. Therefore, our data on hesitancy and ADs apply mainly to these two vaccines. And our survey cannot determine the advantages of different types of vaccines and cannot be used to compare different types of vaccines.

Last, since we used an online questionnaire, we may have missed participants having no or little access to internet.

However, we can infer from our survey data that it is important to inform and educate patients about COVID-19 vaccines, as well as to share knowledge between physicians caring for these patients. The results and regional differences show the need for information, education and common international guidelines to HCPs about COVID-19 vaccination for patients with PID.

## 5 Concluding remarks

A large proportion (41.7%) of the patients denoted hesitancy to COVID-19 vaccination including. More women than men. Furthermore, a significantly larger proportion of the patients living in Eastern Europe reported hesitancy as compared to North America or Western Europe.

The patients self-reported a variety of local and systemic AEs after COVID-19 vaccination with almost one-third of the patients denoting, mostly self-resolving, severe systemic AEs of whom only a minority needed advice from HCPs.

We conclude that an active post-vaccination monitoring for adverse events registered by physicians and immunology nurses is of utmost importance. Our data do not contradict that patients with PID should be advised to receive vaccination against COVID-19 in accordance with applicable national guidelines.

## Data availability statement

The raw data supporting the conclusions of this article will be made available by the authors, without undue reservation.

## Author contributions

All the authors worked on the design of the survey. MP provided the digital version of the questionnaire and insured its translation into different languages (with the support of FH for Dutch and AG for Swedish) and its dissemination. LH provided the analysis of data and prepared figures and tables. All the authors were committed in writing the manuscript, and the modification and revision of the manuscript. All authors approved the submitted version.
